# Neuromonitoring and neuroprotection during neonatal aortic arch surgery: A United Kingdom and Ireland survey

**DOI:** 10.1177/02676591251384585

**Published:** 2025-10-17

**Authors:** William M. McDevitt, Indie Bilkhoo, Edmund Carver, Timothy J. Jones, Stefano Seri, Barney R. Scholefield, Nigel E. Drury

**Affiliations:** 1 Neurophysiology, 156630 Birmingham Children’s Hospital, Birmingham, UK; 2 Cardiovascular Sciences, 1724 University of Birmingham, Birmingham, UK; 3 Clinical Perfusion, 156630 Birmingham Children’s Hospital, Birmingham, UK; 4 Paediatric Anaesthesia, 156630 Birmingham Children’s Hospital, Birmingham, UK; 5 Paediatric Cardiac Surgery, 156630 Birmingham Children’s Hospital, Birmingham, UK; 6 College of Health and Life Sciences, 1722 Aston University, Birmingham, UK; 7 Critical Care Medicine, 7979 The Hospital for Sick Children, Toronto, ON, Canada; 8 Pediatrics, 7938 University of Toronto, Toronto, ON, Canada

**Keywords:** neuroprotection, neuromonitoring, electroencephalography, near-infrared spectroscopy, congenital heart disease, cardiac surgery

## Abstract

**Introduction:**

Neonatal aortic arch surgery is associated with neurological morbidity of varying severity which is detected and potentially limited through neuroprotective strategies. We conducted a survey of healthcare professionals at all neonatal cardiac surgery centres in the United Kingdom and Ireland to determine current intraoperative neuromonitoring and neuroprotection practice.

**Methods:**

An online cross-sectional survey was sent to congenital cardiac surgeons, cardiac anaesthetists, clinical perfusion scientists, and clinical neurophysiology professionals in all 12 level 1 paediatric cardiac surgical centres. Information was sought on their current clinical practice in neonates undergoing aortic arch surgery, including pharmacological management, cardiopulmonary bypass, acid-base and blood pressure management, neuromonitoring, and hypothermic circulatory arrest, and the feasibility and willingness to participate in a future clinical trial of neuroprotective strategies in these patients.

**Results:**

We received 55 (34%) responses, including representatives of all four clinical disciplines in 9 (75%) centres. Cooling to a nasopharyngeal temperature of 18°C before hypothermic circulatory arrest, selective antegrade cerebral perfusion, and near-infrared spectroscopy (NIRS) monitoring are common practice, whereas pharmacology, acid-base management, blood pressure and flow parameters, and NIRS-based interventions vary. In 7 (58%) centres, respondents from all four disciplines were willing to consider participation in a future clinical trial on neuroprotection.

**Conclusions:**

Aspects of intraoperative neuroprotection and neuromonitoring are common across centres, although key areas of practice differ between practitioners and institutions. Most respondents were willing to participate in a future multi-centre clinical trial, which suggests clinical equipoise in the optimal strategy to protect the neonatal brain during aortic arch surgery.

## Introduction

Neuroprotection during cardiac surgery is fundamental to optimise postoperative outcomes for neonates undergoing aortic arch repair. This is, in part, achieved through neuromonitoring, an umbrella term used to describe a range of techniques, devices and strategies to protect the nervous system by detecting and thereby potentially limiting neurological injury. Despite neuroprotective strategies, and neuromonitoring, newly acquired ischaemic brain injury following infant heart surgery is common, and 2–4 times more likely to occur in neonates undergoing aortic arch surgery.^1^ This contributes to the lifelong neurodevelopmental impairment observed in children requiring surgery for congenital heart disease (CHD).^2,3^ There is also marked variability in the frequency of acute surgery-related neurological events between centres,^4^ suggesting that variations in practice may impact on outcomes.

Clinical practice guidelines suggest there are moderate levels of evidence, with varying recommendation of parameters for deep hypothermic circulatory arrest, cerebral perfusion, and anaesthetic management.^5,6^ Other neuroprotective strategies and the utility of neuromonitoring are primarily based on adult studies, laboratory models, or small clinical trials in heterogeneous paediatric populations. As a result, our understanding of optimal protection for the developing neonatal nervous system in those undergoing aortic arch surgery is limited. Current literature highlights variability in neuromonitoring and neuroprotective practices,^7–12^ which may be driven by a lack of evidence to support one technique over another, and lead to outcome variability. The paediatric cardiac surgery literature contains few late-phase, randomised controlled trials (RCT), emphasising the need for high-quality studies to establish best practice,^6,13^ and minimising organ damage during surgery was recently identified as the #1 research priority in children with CHD.^14^

Understanding current neuroprotective and neuromonitoring practices being used across centres provides a foundation for identifying areas that may benefit from standardisation and may lead to improved outcomes. We therefore conducted a survey of current practice during neonatal aortic arch surgery in the UK and Ireland and the willingness of clinicians to randomise patients to an alternative neuromonitoring protocol in the context of a multi-centre clinical trial.

## Methods

In May 2024, a survey link was sent via email to consultant paediatric cardiac surgeons, consultant cardiac anaesthetists, clinical perfusion scientists and healthcare professionals in clinical neurophysiology at all level 1 paediatric cardiac surgery centres in the UK and Ireland. The survey remained open for 8 weeks; non-responders received a follow-up email to prompt completion and were contacted by telephone to encourage at least one response for each specialist group per centre. Respondents provided consent for anonymous reporting of survey data which was collected using REDCap electronic data capture tools.^15^

The study design was informed by a previous survey,^16^ trends in adult aortic neuroprotective practice,^8^ guidelines and recommendations.^6,17^ Participants were asked about their current and routine neuroprotection and neuromonitoring practice in neonates (≤28 days) undergoing aortic arch surgery, not including isolated coarctation repair without cardiopulmonary bypass (CPB), and selected questions were targeted to specific professional groups, where appropriate. Respondents were also asked whether their practice differed with patient age: infants (>28 days-1 year) and older children (1–5 years). Finally, respondents were asked whether they would be willing to adopt an alternative neuromonitoring protocol within the context of a multi-centre clinical trial. The list of questions is shown in Supplemental Table S1.

Data were expressed as counts and percentages or medians with interquartile ranges where appropriate. The relationship between core body temperature, cardiac index (CI), and blood pressure was assessed using Spearman’s correlation coefficients. *p* < .05 was considered significant. Analysis was performed using MATLAB (Mathworks, Natick, MA) and STATA (StataCorp, College Station, TX). The corresponding author had access to all study data and final responsibility for the decision to publish.

## Results

### Respondents

The survey was completed by 55 (34%) healthcare professionals, with responses from 13 (24%) cardiac surgeons, 17 (31%) cardiac anaesthetists, 13 (24%) clinical perfusion scientists, and 12 (22%) neurophysiology professionals (see Supplemental Table S2). At least one professional from each discipline at each centre responded to the survey, except cardiac surgeons with responses from 9 (75%) centres (see Supplemental Table S3).

### Pharmacology

Of 17 anaesthetists, 15 (88%) use a combination of agents to induce general anaesthesia (GA). Nine agents are used during induction, the most common being sevoflurane (14, 82%) and opioids (12, 71%). Anaesthetic maintenance is achieved primarily by isoflurane (11, 65%), and fentanyl for analgesia (13, 77%). Seven (41%) anaesthetists consider certain agents neuroprotective, and three give either steroids, halogenated agents, or magnesium immediately prior to hypothermic circulatory arrest (HCA) to enhance neuroprotection.

### Neuromonitoring

Near-infrared spectroscop (NIRS) is used by respondents in all centres, primarily to guide perfusion strategy (39, 52%) and detect brain injury (29, 39%). Specific NIRS-based interventions varied from checking cannula malposition (33, 14%) to guiding blood transfusion (32, 13%) (Table 1).

**Table 1.** Characteristics of near-infrared spectroscopy (NIRS) and electroencephalography (EEG) use.

Device characteristics	Response, *n* (%)	
	NIRS	EEG
*Utility*	*n* = 75	*n* = 6
Perfusion strategy	39 (52)	0 (0)
Detect injury: Brain	29 (39)	0 (0)
Detect injury: Other organs	4 (5)	0 (0)
Depth of anaesthesia	3 (4)	4 (66)
Prognostication	0 (0)	1 (17)
Seizure detection	0 (0)	1 (17)
*Intervention strategy*	*n* = 232	*n* = 1
Check cannulas	33 (14)	0 (0)
Do blood gas	32 (13)	0 (0)
Transfuse blood	32 (13)	0 (0)
Change temperature management	26 (11)	0 (0)
Vasodilate or vasoconstrict	26 (11)	0 (0)
Adjust ventilation parameters	26 (11)	0 (0)
Adjust cardiac index	24 (10)	0 (0)
Haemoconcentrate	17 (7)	0 (0)
Adjust depth of sedation	10 (4)	1 (100)
Perform ACT test	3 (1)	0 (0)
Bilateral SACP	3 (1)	0 (0)
*Barriers*	*n* = 47	*n* = 79
Lack of equipment	6 (13)	26 (33)
Insufficient evidence	5 (11)	15 (19)
Trained staff	1 (2)	32 (40)
No barriers	35 (74)	6 (8)

ACT: activated coagulation time; SACP: selective antegrade cerebral perfusion.

Electroencephalography (EEG) is ‘sometimes used’ in 5 (42%) centres, predominantly to guide depth of anaesthesia (4, 33%). When asked for specific interventions, EEG is only used to adjust the depth of anaesthesia by one respondent. When asked if the use of NIRS or EEG changed surgical decision making, most respondents said yes to NIRS (32, 74%), and No to EEG (12, 52%). No respondents utilise evoked potentials to monitor the integrity of the spinal cord during surgery.

### Blood and temperature management

**Figure 1.**
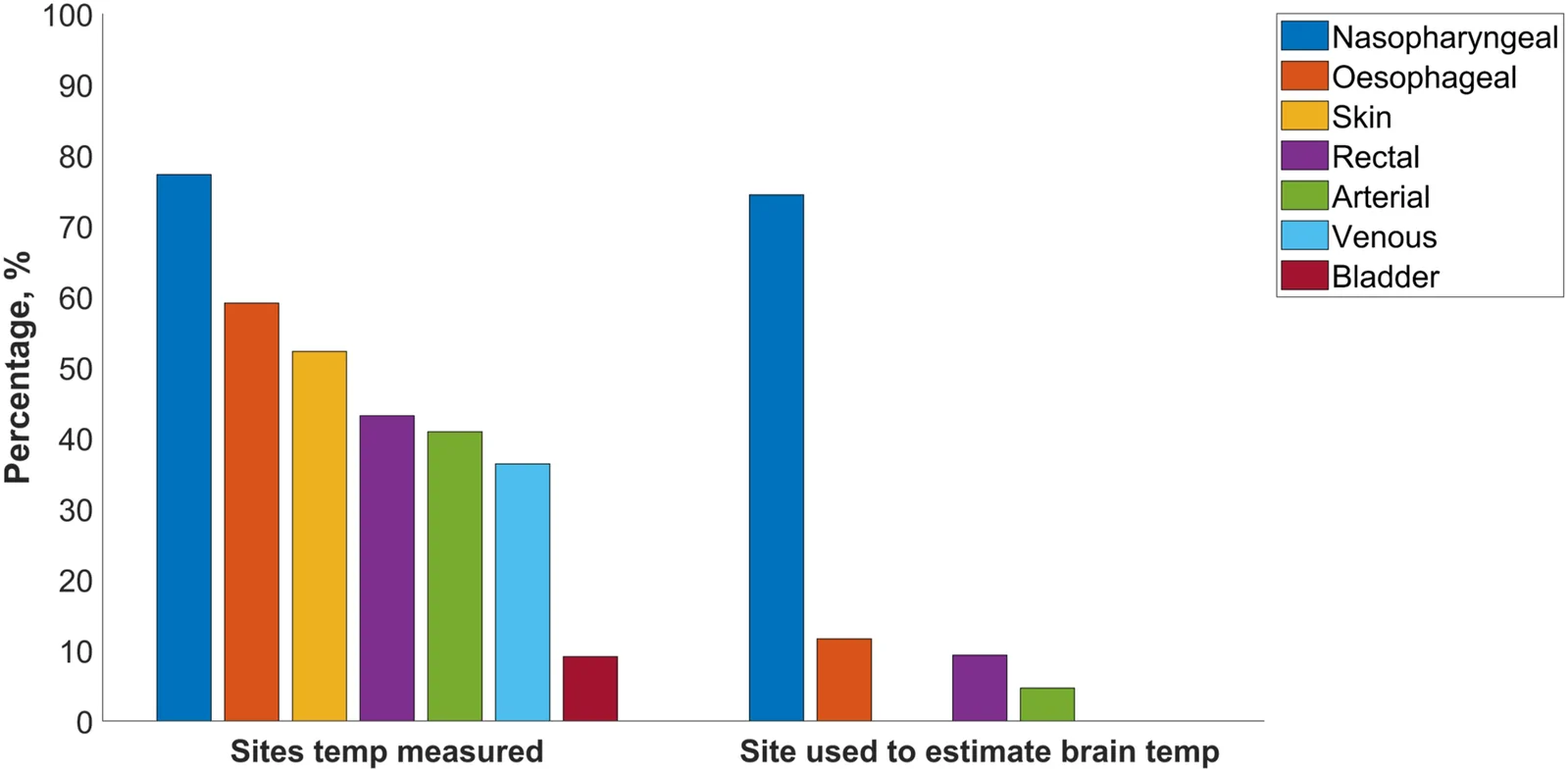
Sites used to measure core body temperature (left) and estimate brain temperature (right).

Most respondents (20, 67%) routinely monitor invasive blood pressure via a combination of unilateral radial and femoral artery catheter probes, and 10 (33%) use either radial or femoral artery blood pressure monitoring in isolation. Blood gas management strategy is heterogenous, with similar numbers of respondents favouring pH-stat (9, 36%) and alpha-stat (7, 28%) alone, or pH-stat during cooling and alpha-stat when rewarming (7, 28%). Two (8%) utilise a blood gas management strategy based on the patient’s core body temperature. The most frequent site used to measure core body temperature is the nasopharynx (32, 73%). Almost all respondents (42, 96%) use multi-site temperature monitoring, the most common being nasopharynx combined with bladder or rectal probes (15, 34%). Most respondents (32, 74%) reported using nasopharyngeal temperature to estimate brain temperature (Figure 1).

### Haemodynamics and cardiopulmonary bypass

**Figure 2.**
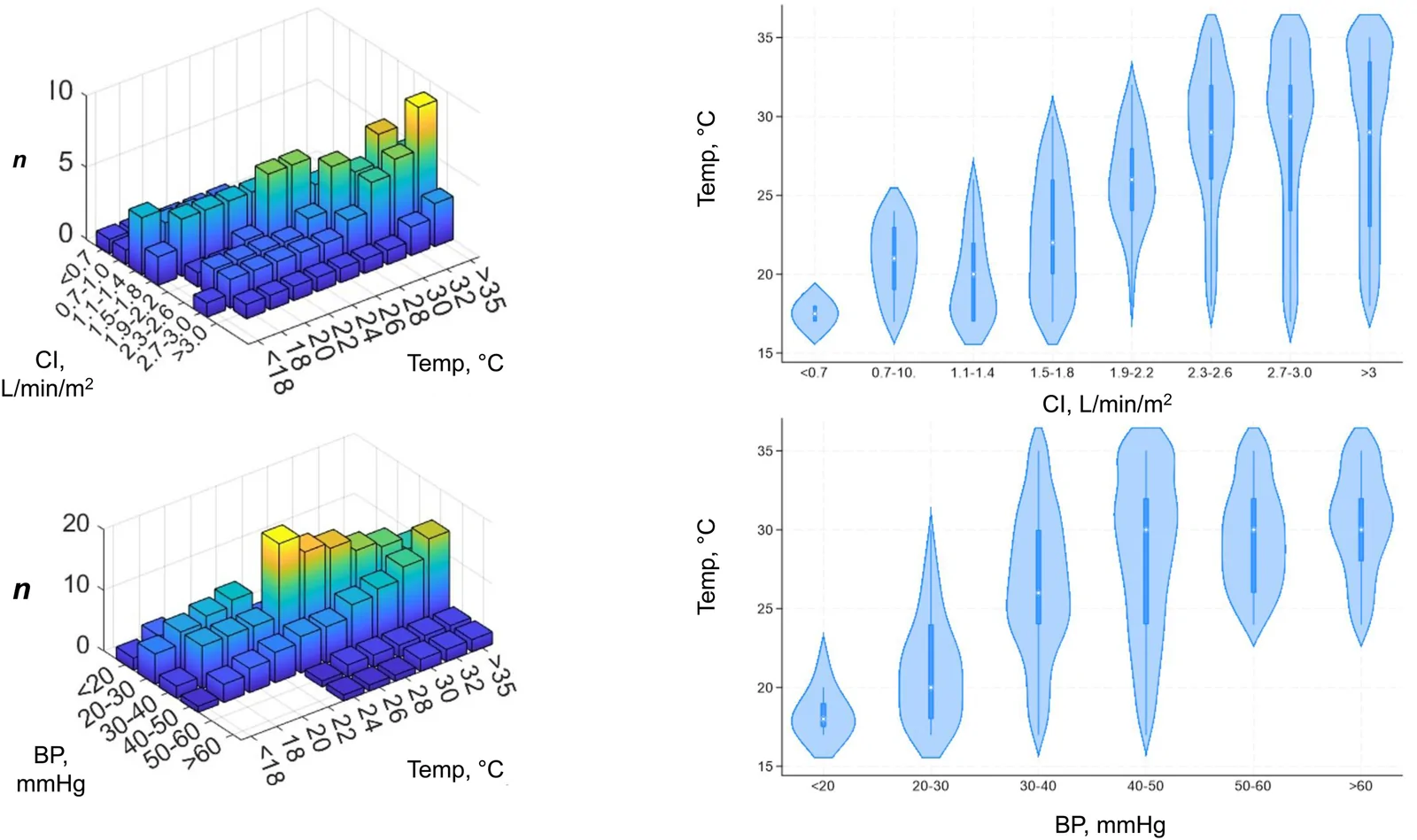
3D histograms illustrating target blood pressure (BP) and cardiac index (CI) used at different core body temperatures (left), with corresponding violin plots (right). The width of each violin indicates number of respondents, the central white dot represents median temperature, thick blue bars indicate interquartile range, and thin blue bars show the range.

In conjunction with the activated coagulation time (ACT) test, 4 (33%) centres use Rotational thromboelastometry and 8 (66%) use Thromboelastogram. Once CPB is established, all centres cool neonates before circulatory arrest. Blood flow (measured as predicted cardiac index, (CI)) and blood pressure targets during cooling varied between respondents (Figure 2). Variability in blood pressure and CI targets increase at lower core body temperatures compared to higher temperatures. Overall, as core body temperature decreases, blood pressure (rs: 0.49, *p*: 0.0001) and CI (rs: 0.54, *p*: <0.0001) targets decrease. During CPB, most respondents (19, 76%) aim for a haematocrit between 25%–30%, however on weaning from CPB, most (20, 80%) raise this target to 31%–35%.

### Hypothermic circulatory arrest

**Figure 3.**
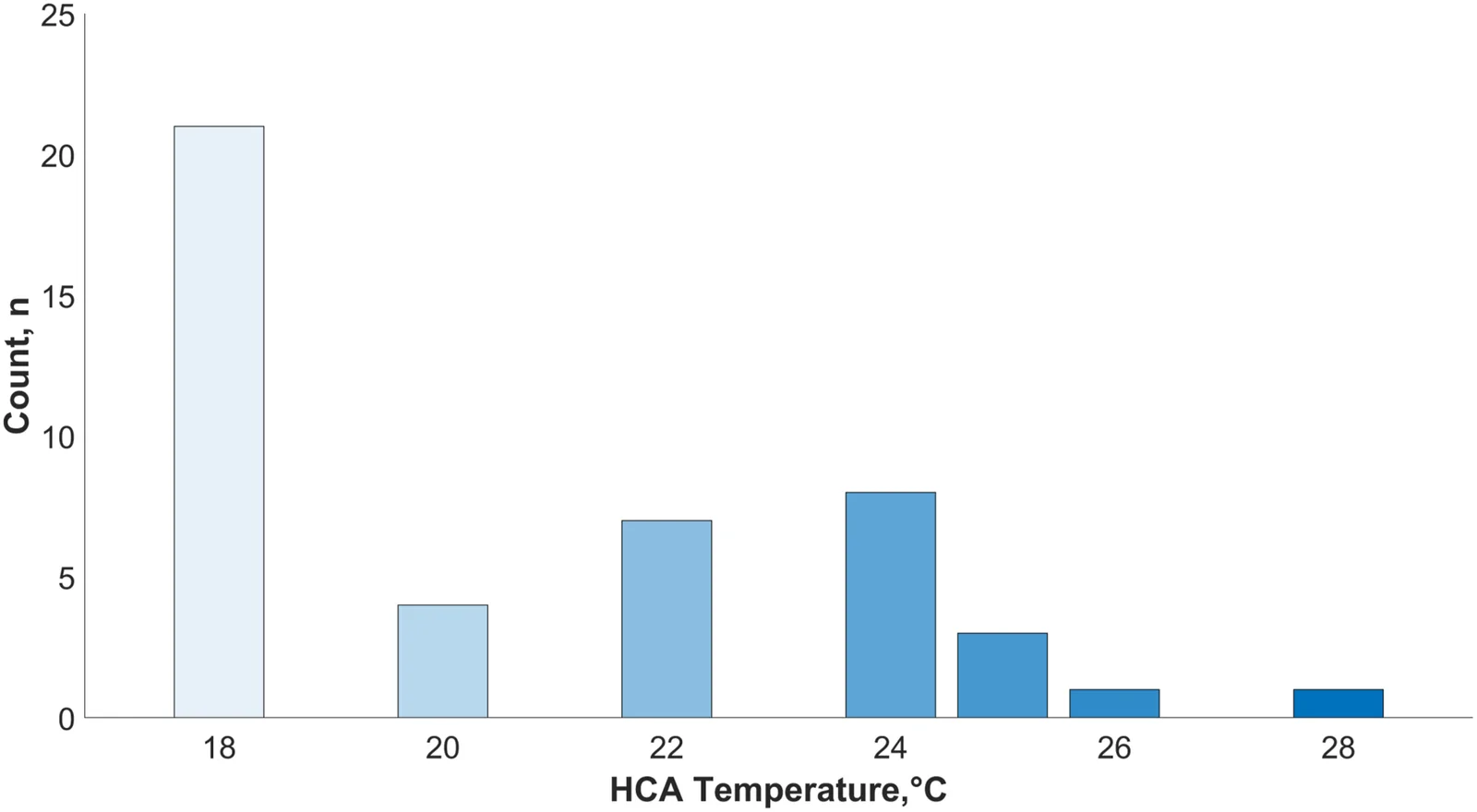
Temperature respondents cool to before hypothermic circulatory arrest (HCA).

Almost half of respondents (21, 48%) across most centres (8, 67%) cool to 18°C as standard practice before HCA (Figure 3), although in the 9 (75%) centres with responses from cardiac surgeons, HCA temperature is not uniform. Twenty-one (47%) respondents report either a fixed period at the target brain temperature, or a minimum cooling duration before HCA. Median cooling duration before HCA is 20 min (range 10–40), whilst one respondent waits for 20 min at 25°C and another waits 10 min at 18°C before starting HCA. Almost all surgeons (12, 92%) utilise selective antegrade cerebral perfusion (SACP) and one (8%) uses HCA without cerebral perfusion. Median temperature of blood delivered via SACP is 20°C (IQR 18–24) and of those who report an SACP blood temperature of 18°C (5, 39%), CI and blood pressure targets range between 0.4 and 1.5 L/min/m^2^ and <20–60 mmHg, respectively.

### Neurological injury and future research

With these temperature and perfusion strategies, 10 (77%) cardiac surgeons from 8 (67%) centres provided a duration of HCA after which they would be concerned about neonatal brain injury (median 40 min, range 20–100). In 8 (67%) centres, CT, MRI, and ultrasound were routinely used to detect brain injury and in 4 (33%) centres, NIRS and EEG were used where clinically relevant. Within the context of a future multi-centre clinical trial of a novel neuromonitoring protocol, 7 (58%) centres had at least one cardiac surgeon, anaesthetist, clinical perfusionist and professional within clinical neurophysiology that would be willing to participate, depending on what would be required.

## Discussion

In this survey of current practice in the UK and Ireland, we found that aspects of intraoperative neuroprotection and neuromonitoring are common across centres, although key areas of practice differ between practitioners and institutions. As no respondents use evoked potentials to monitor the integrity of the spinal cord, our findings primarily relate to monitoring and protection of the neonatal brain. Almost half of respondents cool to a nasopharyngeal temperature of 18°C before HCA, and almost all use SACP, NIRS monitoring, and ancillary tests to assess coagulation during CPB. In principle, respondents from most centres were interested in participating in a multi-centre clinical trial of neuromonitoring during neonatal aortic arch surgery, suggesting that there is clinical equipoise in the optimal neuromonitoring and neuroprotection strategy.

### Pharmacology

The neuroprotective benefit of certain agents used during neonatal cardiac surgery has been the subject of previous research.^18–22^ Animal studies demonstrate that anaesthetic agents can induce changes in the immature central nervous system changes, with long-term effects on neurodevelopmental outcome.^23–25^ While no evidence links adverse cognitive development to short-duration, single anaesthetic agent exposure, the effect of prolonged exposure to multiple agents is unknown.^26^

The nature of cardiac surgery requires prolonged exposure to these agents. The majority (88%) of cardiac anaesthetist respondents use a combination of agents during GA induction, and all use multiple agents during GA maintenance for prolonged periods. Although trends were seen in agent preference, our findings reflect the lack of evidence for one anaesthetic technique over another to impact on neurological outcome. For example, steroids demonstrate neuroprotective characteristics in animal studies,^27^ but to date, this strategy has not been proven to have widespread clinical benefit^28^ although there may be some benefit in selected sub-groups, such as those undergoing palliative procedures.^29^ However, only 10% of respondents consider steroids neuroprotective and 4% administer them prior to HCA, presumably because there are no large-scale RCTs demonstrating clinical benefit.^6^

### Blood pressure and flow

Two thirds of respondents monitor invasive upper and lower limb blood pressure. Although neonatal arch hypoplasia is seen in isolation or in combination with more severe CHD, and therefore blood pressure monitoring techniques vary accordingly, a combination of upper and lower limb monitoring helps track cerebral and descending aortic perfusion. It may also help to detect a gradient across the proximal aorta and aortic arch,^6^ which can prompt intervention.

The literature presents conflicting findings on blood flow characteristics during hypothermia, and significant variability between patients.^30,31^ Cerebral blood flow velocity during CPB-induced cooling has been shown to increase up until the point of HCA, at temperatures as cold as 20°C.^32^ At temperatures below 20°C, it is generally considered that cerebral autoregulation is lost, and as a result careful adjustments to CI and blood pressure targets should be made, reflected by the CI and blood pressure management strategies reported in our survey. In Figure 2, there is a clear relationship between CI and blood pressure targets during CPB-assisted cooling; as temperature decreases, so too does CI and blood pressure, although variability in CI and blood pressure targets at colder temperatures increased.

### Haematocrit and coagulation

Haematocrit and coagulation practices are relatively uniform across survey respondents. Although research in this field is relatively scarce, aiming for 25%–30% haematocrit whilst on CPB,^33,34^ and higher when weaning from CPB has been associated with more favourable outcomes.^35^ This practice appears widespread in the UK and Ireland, although few aim for >35% haematocrit during CPB weaning. Higher post-CPB haematocrit makes blood more viscous, facilitating haemostasis and reducing the likelihood of blood product transfusion. However, blood transfusion to counter haemodilution is not without risk and requires manipulation of other blood-based parameters, such as acid-base management.^36^

### Acid-base management

We found a near-equal split in acid-base management strategies, in line with current literature and suggesting a lack of conclusive evidence for one strategy over another.^6^ In animal models, procedures involving deep HCA and a pH-stat strategy were associated with improved postoperative neurology.^37^ In infants, pH-stat management was associated with lower postoperative morbidity, shorter EEG recovery time, and shorter duration of intubation and ITU stay, but no differences in long term neurological outcomes.^38^

### Temperature

Previous surveys of adult neuroprotective practice have described similar results to our survey regarding the use of SACP, NIRS, and HCA.^8^ Most adult aortic centres cool to 22°C–26°C before HCA, whilst our survey shows that 18°C is more common in neonates. Cooling reduces cerebral metabolism in a dose-dependant fashion, with inter- and intra-patient variability in the degree to which metabolism changes during stages of hypothermia.^39^ The lack of a uniform strategy for the management of patients subjected to cooling during surgery may explain the variability seen in adult cooling strategies^31^ and why some survey respondents prefer warmer temperatures before HCA.

### HCA and cerebral perfusion

Adult guidelines report moderate hypothermia with SACP as potentially the safest cerebral protection strategy for total arch replacement.^40^ In neonates undergoing isolated arch repair, cerebral perfusion or limited duration DHCA are considered reasonable strategies for optimising neurological outcome,^5^ whilst in the Norwood operation, limiting DHCA duration, using intermittent cerebral perfusion for prolonged periods of arrest, and low-flow or selective cerebral perfusion are commonplace.^6^

A recent study reported that in over 24,000 neonatal cardiac procedures, the use of cerebral perfusion was associated with lower risk of neurological injury, and authors comment that practice variation may adversely impact outcomes.^41^ There were no associations between temperature, cerebral perfusion, and neurological injury in those undergoing isolated aortic arch procedures, perhaps due to the relatively lower number of these procedures performed and relatively low incidence of neurological injury. However, the risk of neurological injury during the Norwood procedure was higher with normothermia compared with deep hypothermia when temperature was analysed as a categorical variable, and lower when cerebral perfusion was used.

In North America, more than 80% of cardiac surgeons use cerebral perfusion post HCA,^42^ similar to the 92% in our UK and Ireland survey. Although popular, the evidence base for enhanced neuroprotection with cerebral perfusion is limited, and lacks detail regarding optimal CI, blood temperature and pressure, acid-base management, and HCA temperature.^43^ This suggests the evidence base may benefit from studies that incorporate neuromonitoring to investigate optimal neuroprotective HCA strategies.

### Neuromonitoring

In a recent scoping review of neuromonitoring practice in neonates with CHD,^7^ intraoperative NIRS and EEG were frequently used, but it remains unclear whether NIRS- or EEG-based intervention is associated with improved outcome. Adult aortic guidelines recommend intervention based on NIRS monitoring,^40^ and although recommended as part of anaesthetic management for infants undergoing Norwood palliation,^6^ there does not appear to be consensus on what interventions should be triggered. This is in keeping with survey findings, and the rate at which NIRS is used in the UK and Ireland is similar to European centres.^11^ All centres utilise NIRS but perform a range of interventions. This contrasts with EEG, where few use it but of those that do, its utility is more apparent.

EEG monitoring is not fully utilised in neonatal cardiac surgery due to a lack of trained staff, equipment, and evidence demonstrating utility. EEG can be used guide intraoperative cerebral protection,^10^ prognose outcome^44^ and detect postoperative seizures^45^ but more evidence is needed to demonstrate whether intraoperative EEG-based intervention improves postoperative outcomes.

### Limitations

Our overall response rate of 34% is similar to other surveys in this field^8^ but included at least one response from each of the professional groups across 75% of surgical centres in UK and Ireland. This survey reflects the opinions and behaviours of individuals in UK and Ireland centres and may not represent the uniform practice of those centres. We did not ask whether responses were based on departmental protocols or the literature, so our findings reflect self-reported practice, and no inferences about clinical efficacy should be drawn. However, clear commonalities and disparities between respondents and centres are apparent, which warrants further investigation. Some aspects of management may also have differed according to patient factors, such as the complexity of CHD, which may not have been captured, even with the availability of free text responses. This survey is cross-sectional and cannot capture changes in practice over time. Although questions asked of respondents were comprehensive and in line with current literature recommendations, there are aspects of perioperative neuroprotection and neuromonitoring that were not considered, and there was limited scope for respondents to elaborate on chosen responses. To address this, we will interview respondents interested in participating in a multi-centre RCT to further explore their willingness to change practice within a trial.

## Conclusions

In summary, aspects of neuroprotection and neuromonitoring during neonatal aortic arch surgery such as cooling to a nasopharyngeal temperature of 18°C before HCA, and the use of SACP, NIRS monitoring, and ancillary tests to assess coagulation during CPB, are similar across centres. However, there is heterogeneity in key aspects of practice and most respondents were willing to participate in a future multi-centre clinical trial, which suggests clinical equipoise in the optimal strategy to protect the neonatal brain during aortic arch surgery.

## Supplemental Material

Supplemental Material - Neuromonitoring and neuroprotection during neonatal aortic arch surgery: A United Kingdom and Ireland survey Supplemental Material for Neuromonitoring and neuroprotection during neonatal aortic arch surgery: A United Kingdom and Ireland survey by William M. McDevitt, Indie Bilkhoo, Edmund Carver, Timothy J. Jones, Stefano Seri, Barney R. Scholefield, Nigel E. Drury in Perfusion

Supplemental Material - Neuromonitoring and neuroprotection during neonatal aortic arch surgery: A United Kingdom and Ireland survey Supplemental Material for Neuromonitoring and neuroprotection during neonatal aortic arch surgery: A United Kingdom and Ireland survey by William M. McDevitt, Indie Bilkhoo, Edmund Carver, Timothy J. Jones, Stefano Seri, Barney R. Scholefield, Nigel E. Drury in Perfusion

## Data Availability

Data are available in online supplementary material and via the corresponding author on request.*
