# Supplementary material for: Neuromonitoring and neuroprotection during neonatal aortic arch surgery: A United Kingdom and Ireland survey
Source: Perfusion. 2025 Oct 17;41(6):957–65. doi: 10.1177/02676591251384585 (PMC13490662; doi:10.1177/02676591251384585)
Supplement: Supplementary file 1 [file sj-pdf-1-prf-10.1177_02676591251384585.pdf]

Neuromonitoring and neuroprotection during neonatal aortic arch surgery: a UK & Ireland survey

We are conducting this survey of current neuromonitoring and neuroprotective practice during neonatal aortic arch surgery in the UK & Ireland to identify if there are variations in practice and willingness to adopt a neuromonitoring protocol within the context of a proposed multi-centre randomised controlled trial.

This survey will take approximately 10-15 minutes to complete. You may save & return later by clicking the button at the end of this survey, then follow the link in the email or click 'Returning?' at the top right of this page and enter your individual return code.

The survey will close on Friday 19th July 2024. Your participation is entirely voluntary and you can withdraw at any time by emailing: w.mcdevitt@nhs.net. You can find out what we do with your data in our Privacy Notice.

Please enter your name: ________________________________

At which paediatric cardiac surgery centre do you work?

- ◯ Birmingham - Children's Hospital
- ◯ Bristol - Royal Hospital for Children
- ◯ Dublin - Our Lady's Children's Hospital
- ◯ Glasgow - Royal Hospital for Children
- ◯ Leeds - General Infirmary
- ◯ Leicester - Glenfield Hospital
- ◯ Liverpool - Alder Hey Children's Hospital
- ◯ London - Evelina London Children's Hospital
- ◯ London - Great Ormond Street Hospital for Children
- ◯ London - Royal Brompton Hospital
- ◯ Newcastle - Freeman Hospital
- ◯ Southampton - Wessex Cardiothoracic Centre

What is your role?

- ◯ Paediatric Cardiac Surgeon
- ◯ Clinical Perfusionist
- ◯ Paediatric Cardiac Anaesthetist
- ◯ Neurophysiologist- Clinical Neurophysiologist or Clinical Scientist

The following questions relate to your routine practice in the management of NEONATES undergoing aortic arch surgery. There are separate questions at the end of this survey relating to any differences in practice for infants and older children.

Which of the following do you routinely give at anaesthetic induction? Tick all that apply.

- ☐ Isoflurane
- ☐ Sevoflurane
- ☐ Morphine
- ☐ Midazolam
- ☐ Fentanyl
- ☐ Ketamine
- ☐ Clonidine
- ☐ Propofol
- ☐ Nitrous oxide
- ☐ Dexmedetomidine
- ☐ Thiopental
- ☐ Other

Other - please provide details: ________________________________

What do you routinely use to maintain anaesthesia? Tick all that apply.	☐ Isoflurane<br>☐ Sevoflurane<br>☐ Propofol<br>☐ Midazolam<br>☐ Dexmedetomidine<br>☐ Other
Other - please provide details:	__________________________________
What do you routinely use to provide analgesia? Tick all that apply.	☐ Morphine<br>☐ Fentanyl<br>☐ Remifentanil<br>☐ Dexmedetomidine<br>☐ Other
Other - please provide details:	__________________________________
Which of the following pharmacological agents do you consider to be neuroprotective when used during neonatal aortic arch surgery with deep hypothermic circulatory arrest? Tick all that apply.	☐ Inhalational anaesthesia<br>☐ Total intravenous anaesthesia<br>☐ Sodium Nitroprusside<br>☐ Thiopental<br>☐ Ketamine<br>☐ Allopurinol<br>☐ Phentolamine<br>☐ Corticosteroids<br>☐ Dextromethorphan<br>☐ Nicardipine<br>☐ Mannitol<br>☐ Erythropoietin<br>☐ Other
Other - please provide details:	__________________________________
Do you administer any of the following pharmacological agents IMMEDIATELY prior to hypothermic circulatory arrest to have a neuroprotective effect? Tick all that apply.	☐ Thiopental<br>☐ Corticosteroids<br>☐ Ketamine<br>☐ Allopurinol<br>☐ Phentolamine<br>☐ Dextromethorphan<br>☐ Nicardipine<br>☐ Mannitol<br>☐ Erythropoietin<br>☐ Midazolam<br>☐ Nitrous oxide<br>☐ Isoflurane<br>☐ Sevoflurane<br>☐ Propofol<br>☐ Morphine<br>☐ Fentanyl<br>☐ Remifentanil<br>☐ Dexmedetomidine<br>☐ Sodium Nitroprusside<br>☐ I do not administer any pharmacological agent immediately prior to hypothermic circulatory arrest<br>☐ Other
Other - please provide details:	__________________________________

What sites/methods do you use routinely to measure blood pressure during neonatal aortic arch surgery? Tick all that apply.

- ☐ Non-invasive
- ☐ Invasive radial artery - unilateral
- ☐ Invasive radial artery - bilateral
- ☐ Invasive femoral artery
- ☐ Other

Other - please provide details:

____

Which of the following blood pH management strategies do you routinely use during neonatal aortic arch surgery whilst on CPB? Tick all that apply.

- ☐ Alpha stat only
- ☐ pH stat only
- ☐ pH stat during cooling, Alpha stat during rewarming,
- ☐ Alpha stat during cooling, pH stat during rewarming,
- ☐ Other

Other - please provide details:

____

Do you routinely use Near Infrared Spectroscopy (NIRS) monitoring during neonatal aortic arch surgery?

- ◯ Yes
- ◯ No
- ◯ Sometimes

Sometimes - please provide details:

____

What sites do you use routinely for NIRS monitoring during neonatal aortic arch surgery? Tick all that apply.

- ☐ Unilateral forehead/brain
- ☐ Bilateral forehead/brain
- ☐ Unilateral flank/abdomen/kidney- during
- ☐ Bilateral flank/abdomen/kidney- during
- ☐ Don't know
- ☐ Other

Other - please provide details:

____

What do you use NIRS monitoring for during neonatal aortic arch surgery? Tick all that apply.

- ☐ Perfusion strategy
- ☐ Depth of anaesthesia
- ☐ Brain injury detection
- ☐ Visceral organ injury detection
- ☐ Other

Other - please provide details:

____

Would you change your surgical/perfusion strategy based on real-time NIRS values?

- ◯ Yes
- ◯ No
- ◯ No, but I would consider it alongside other measures of potential organ injury
- ◯ Other

Other - please provide details:

____

Which of the following would you routinely perform based on NIRS values? Tick all that apply.	☐ Do a blood gas<br>☐ Adjust ventilation parameters<br>☐ Check cannulas<br>☐ Do an ACT test<br>☐ Adjust cardiac index<br>☐ Administer a vasoconstrictor/vasodilator<br>☐ Bilateral carotid cannulation for cerebral perfusion<br>☐ Blood transfusion<br>☐ Change in temperature management<br>☐ Adjust depth of sedation<br>☐ Haemoconcentrate<br>☐ Other
Other - please provide details:	__________________________________
What are the barriers to NIRS monitoring during neonatal aortic arch surgery at your institution? Tick all that apply.	☐ Lack of evidence to support its utility<br>☐ Lack of experienced staff to interpret real-time data<br>☐ Lack of NIRS equipment/funding<br>☐ There are no barriers to NIRS monitoring<br>☐ Other
Other - please provide details:	__________________________________
Do you routinely use electroencephalography (EEG) monitoring during neonatal aortic arch surgery?	◯ Yes<br>◯ No<br>◯ Sometimes
Sometimes - please provide details:	__________________________________
What type of EEG is used? Tick all that apply.	☐ Processed/quantitative<br>☐ Unprocessed/raw<br>☐ Don't know<br>☐ Other
Other- please provide details	__________________________________
What EEG electrode sites do you use routinely for monitoring during neonatal aortic arch surgery?	☐ Forehead only<br>☐ Limited scalp (between 4-12 electrodes)<br>☐ Full scalp (>12 electrodes)<br>☐ Don't know<br>☐ Other
Other - please provide details:	__________________________________
What do you use EEG monitoring for during neonatal aortic arch surgery? Tick all that apply.	☐ Depth of anaesthesia<br>☐ Rate of cooling<br>☐ Seizure detection/management<br>☐ Hypothermic circulatory arrest strategy<br>☐ Rate of rewarming<br>☐ Prognostication<br>☐ Other

Other - please provide details:	__________________________________
Would you change your surgical/perfusion strategy based on real-time EEG findings?	◯ Yes<br>◯ No<br>◯ No, but I would consider it alongside other measures of potential organ injury<br>◯ Other
Other - please provide details:	__________________________________
Which of the following would you routinely perform based on EEG? Tick all that apply.	☐ Do a blood gas<br>☐ Adjust ventilation parameters<br>☐ Check cannulas<br>☐ Do an ACT test<br>☐ Adjust cardiac index<br>☐ Administer a vasoconstrictor/vasodilator<br>☐ Bilateral carotid cannulation for cerebral perfusion<br>☐ Blood transfusion<br>☐ Change in temperature management<br>☐ Adjust depth of sedation<br>☐ Haemoconcentrate<br>☐ Other
Other - please provide details:	__________________________________
What are the barriers to EEG monitoring during neonatal aortic arch surgery at your institution? Tick all that apply.	☐ Lack of evidence to supporting its utility<br>☐ Lack of experienced staff to interpret real-time data<br>☐ Lack of EEG equipment/funding<br>☐ There are no barriers to EEG monitoring<br>☐ Other
Other - please provide details:	__________________________________
Do you routinely use motor evoked potential (MEP) or somatosensory evoked potential (SSEP) monitoring during neonatal aortic arch surgery?	◯ Yes<br>◯ No<br>◯ Sometimes
Sometimes - please provide details:	__________________________________

Whilst on cardiopulmonary bypass (CPB), what is your cardiac index (L/min/m2) maintained at for the following brain temperatures? Tick all that apply.

(Tick "NA" if listed temperature is not achieved during neonatal aortic arch surgery).

NB. Options for this question will only appear for Perfusionists.

	< 0.7	0.7 - 1.0	1.1 - 1.4	1.5 - 1.8	1.9 - 2.2	2.3 - 2.6	2.7 - 3.0	>3.0	NA

>35°C	☐	☐	☐	☐	☐	☐	☐	☐	☐
32°C	☐	☐	☐	☐	☐	☐	☐	☐	☐
30°C	☐	☐	☐	☐	☐	☐	☐	☐	☐
28°C	☐	☐	☐	☐	☐	☐	☐	☐	☐
26°C	☐	☐	☐	☐	☐	☐	☐	☐	☐
24°C	☐	☐	☐	☐	☐	☐	☐	☐	☐
22°C	☐	☐	☐	☐	☐	☐	☐	☐	☐
20°C	☐	☐	☐	☐	☐	☐	☐	☐	☐
18°C	☐	☐	☐	☐	☐	☐	☐	☐	☐
< 18°C	☐	☐	☐	☐	☐	☐	☐	☐	☐

Whilst on cardiopulmonary bypass (CPB), what is the arterial blood pressure (mmHg) maintained at for the following brain temperatures? Tick all that apply.

Tick "NA" if listed temperature is not achieved during neonatal aortic arch surgery.

NB. Options for this question will only appear for Cardiac Surgeons and Perfusionists.

	< 20	20 - 30	30 - 40	40 - 50	50 - 60	>60	NA
>35°C	☐	☐	☐	☐	☐	☐	☐
32°C	☐	☐	☐	☐	☐	☐	☐
30°C	☐	☐	☐	☐	☐	☐	☐
28°C	☐	☐	☐	☐	☐	☐	☐
26°C	☐	☐	☐	☐	☐	☐	☐
24°C	☐	☐	☐	☐	☐	☐	☐
22°C	☐	☐	☐	☐	☐	☐	☐
20°C	☐	☐	☐	☐	☐	☐	☐
18°C	☐	☐	☐	☐	☐	☐	☐
< 18°C	☐	☐	☐	☐	☐	☐	☐

What sites do you use routinely to measure temperature continuously during neonatal aortic arch surgery? Tick all that apply.

☐ Oesophageal
☐ Nasopharyngeal
☐ Bladder
☐ Skin
☐ Rectal
☐ Arterial blood
☐ Venous blood
☐ Other

Other - please provide details:

____

Of these sites, which is your preferred option to estimate BRAIN temperature during neonatal aortic arch surgery?

◯ Oesophageal
◯ Nasopharyngeal
◯ Bladder
◯ Skin
◯ Rectal
◯ Arterial blood
◯ Venous blood
◯ Other

Other - please provide details:	__________________________________
To what BRAIN temperature (°C) do you routinely cool prior to circulatory arrest in neonates undergoing aortic arch surgery?	13°C 25°C 36°C<br>(Place a mark on the scale above)
What patient factors may change your target brain temperature prior to circulatory arrest, and if so, how?	__________________________________
Do you have a minimum cooling duration prior to circulatory arrest?	◯ Yes, a minimum cooling duration before circulatory arrest<br>◯ Yes, a fixed amount of time at the target brain temperature<br>◯ Yes, both a minimum cooling duration and a fixed amount of time at the target brain temperature<br>◯ No<br>◯ Other
Other - please provide details:	__________________________________
'Minimum cooling duration before circulatory arrest' - please provide total cooling duration before circulatory arrest in minutes.	0 mins 50 mins 100 mins<br>(Place a mark on the scale above)
'Fixed amount of time at the target brain temperature' - please provide the total duration at this temperature before circulatory arrest in minutes.	0 mins 50 mins 100 mins<br>(Place a mark on the scale above)
During hypothermic circulatory arrest, which of the following best reflects your routine perfusion practice for neonatal aortic arch surgery?	◯ Hypothermia alone<br>◯ Regional low flow perfusion (Lf-CPB)<br>◯ Retrograde cerebral perfusion (RCP)<br>◯ Selective antegrade cerebral perfusion (SACP)<br>◯ Other
Other - please provide details:	__________________________________

What is your cardiac index (L/min/m2) maintained at during the following perfusion strategies. Please tick all that apply.

(Please select "NA" if this perfusion strategy is not applicable to your practice).

NB. Options for this question will only appear for Perfusionists.

	< 0.4	0.4 - 0.7	0.8 - 1.1	1.2 - 1.5	1.6 - 1.9	2.0 - 2.3	2.4 - 2.7	2.8 - 3.1	>3.1	NA

SACP	☐	☐	☐	☐	☐	☐	☐	☐	☐	☐
RCP	☐	☐	☐	☐	☐	☐	☐	☐	☐	☐
No circulatory arrest, just Lf-CPB	☐	☐	☐	☐	☐	☐	☐	☐	☐	☐

What is your cerebral arterial blood pressure (mmHg) maintained at during the following perfusion strategies. Please tick all that apply.

(Please select "NA" if this perfusion strategy is not applicable to your practice).

NB. Options for this question will only appear for Perfusionists.

	< 20	20-30	30-40	40-50	50-60	>60	NA
SACP	☐	☐	☐	☐	☐	☐	☐
RCP	☐	☐	☐	☐	☐	☐	☐
No circulatory arrest, just Lf-CPB	☐	☐	☐	☐	☐	☐	☐

What is your cerebral blood temperature (°C) maintained at as part of your cerebral perfusion strategy?

10°C 20°C 30°C

(Place a mark on the scale above)

With this temperature and perfusion strategy, what duration of hypothermic circulatory arrest would neonatal brain injury start to be a concern for you?

0 mins 50 mins 100 mins

(Place a mark on the scale above)

Which of the following perioperative devices are routinely used in your centre to diagnose neurological injury in neonates undergoing aortic arch surgery? Tick all that apply.

☐ MRI brain
☐ CT brain
☐ Ultrasound (US) brain
☐ NIRS
☐ EEG- unprocessed, or raw EEG
☐ qEEG- processed (amplitude integrated EEG and/or spectral arrays)
☐ Evoked potentials (SSEP/MEP)
☐ Transcranial Doppler (TCD)
☐ Blood biomarkers (NSE, S-100B)
☐ Other

Other - Please provide details:

____

During neonatal aortic arch surgery, what haematocrit do you aim for WHILST ON CPB?

◯ Haematocrit < 15%
◯ Haematocrit 15-20%
◯ Haematocrit 21-24%
◯ Haematocrit 25-30%
◯ Haematocrit 30-35%
◯ >35%
◯ Other

Other - please provide details:

____

During neonatal aortic arch surgery, what haematocrit do you aim for WHEN COMING OFF CPB?	◯ Haematocrit < 15%<br>◯ Haematocrit 15-20%<br>◯ Haematocrit 21-24%<br>◯ Haematocrit 25-30%<br>◯ Haematocrit 30-35%<br>◯ >35%<br>◯ Other
Other - please provide details:	__________________________________
In addition to activated coagulation time (ACT), which of the following tests do you use to guide heparin anticoagulation and products given after CPB? Tick all that apply.	☐ TEG- Hertert thromboelastography<br>☐ ROTEM- Rotational thromboelastometry<br>☐ None, ACT only<br>☐ Other
Other - please provide details:	__________________________________
Do you have point of care Full Blood Count (FBC) in your centre?	◯ Yes<br>◯ No

The following questions relate to how your practice changes for aortic arch surgery for infants (31 days old - 1 year old) and older children (> 1 year old - 5 years old)

Does your practice for INFANTS (31 days old - 1 year) differ from that described above for neonates?	◯ Yes<br>◯ No
How does it differ for infants? Please provide details:	____
Does your practice for OLDER CHILDREN (> 1 year - 5 years) differ from that described above for neonates?	◯ Yes<br>◯ No
How does it differ for older children? Please provide details	____

Finally, we are interested in assessing barriers and facilitators to a neuromonitoring protocol to be used in a potential multi-centre randomised controlled trial in neonates and infants undergoing aortic arch surgery.

In principle, would you be interested in participating in a multi-centre trial of neuromonitoring during paediatric aortic arch surgery?	◯ Yes<br>◯ No<br>◯ Possibly, depending on what is required
If you are happy for us to contact you regarding future research in this area, please provide your email address:	__________________________________
