# Supplementary material for: Neuromonitoring and neuroprotection during neonatal aortic arch surgery: A United Kingdom and Ireland survey
Source: Perfusion. 2025 Oct 17;41(6):957–65. doi: 10.1177/02676591251384585 (PMC13490662; doi:10.1177/02676591251384585)
Supplement: Supplementary file 2 [file sj-pdf-2-prf-10.1177_02676591251384585.pdf]

Table S1. Survey questions and which specialists were invited to answer each question.

No	Survey question	Specialists invited to answer each survey question			
		Surgeons	Anaesthetists	Perfusionists	Neurophysiologists
1	Please enter your name	Yes	Yes	Yes	Yes
2	At which paediatric cardiac surgery centre do you work?	Yes	Yes	Yes	Yes
3	What is your role?	Yes	Yes	Yes	Yes
4	Which of the following do you routinely give at anaesthetic induction? Tick all that apply.	No	Yes	No	No
5	What do you routinely use to maintain anaesthesia? Tick all that apply.	No	Yes	No	No
6	What do you routinely use to provide analgesia? Tick all that apply.	No	Yes	No	No
7	Which of the following pharmacological agents do you consider to be neuroprotective when used during neonatal aortic arch surgery with deep hypothermic circulatory arrest? Tick all that apply	No	Yes	No	No
8	Do you administer pharmacological agents IMMEDIATELY prior to hypothermic circulatory arrest to have a neuroprotective effect? Tick all that apply	No	Yes	No	No
9	What sites/methods do you use routinely to measure blood pressure during neonatal aortic arch surgery? Tick all that apply.	Yes	Yes	No	No
10	Which of the following blood pH management strategies do you routinely use during neonatal aortic arch surgery whilst on CPB? Tick all that apply	No	Yes	Yes	No
11	Do you routinely use Near Infrared Spectroscopy (NIRS) monitoring during neonatal aortic arch surgery?	Yes	Yes	Yes	Yes
12	What sites do you use routinely for NIRS monitoring during neonatal aortic arch surgery? Tick all that apply.	No	Yes	No	No
13	What do you use NIRS monitoring for during neonatal aortic arch surgery? Tick all that apply.	Yes	Yes	Yes	Yes
14	Would you change your surgical/perfusion strategy based on real-time NIRS values?	Yes	Yes	Yes	Yes
15	Which of the following would you routinely perform based on NIRS values? Tick all that apply.	Yes	Yes	Yes	Yes
16	What are the barriers to NIRS monitoring during neonatal aortic arch surgery at your institution? Tick all that apply	Yes	Yes	Yes	Yes
17	Do you routinely use electroencephalography (EEG) monitoring during neonatal aortic arch surgery?	Yes	Yes	Yes	Yes

18	What type of EEG is used? Tick all that apply.	No	Yes	No	Yes
19	What EEG electrode sites do you use routinely for monitoring during neonatal aortic arch surgery? Please tick all that apply	No	Yes	No	Yes
20	What do you use EEG monitoring for during neonatal aortic arch surgery? Tick all that apply	Yes	Yes	Yes	Yes
21	Would you change your surgical/perfusion strategy based on real-time EEG findings ?	Yes	Yes	Yes	Yes
22	Which of the following would you routinely perform based on EEG? Tick all that apply	Yes	Yes	No	Yes
23	What are the barriers to EEG monitoring during neonatal aortic arch surgery at your institution? Tick all that apply	Yes	Yes	Yes	Yes
24	Do you routinely use motor evoked potential (MEP) or somatosensory evoked potential (SSEP) monitoring during neonatal aortic arch surgery?	Yes	No	No	Yes
25	Whilst on cardiopulmonary bypass (CPB), what is your cardiac index (L/min/m2) maintained at for the following brain temperatures? Tick all that apply. (Tick "NA" if listed temperature is not achieved during neonatal aortic arch surgery).	No	No	Yes	No
26	Whilst on cardiopulmonary bypass (CPB), what is the arterial blood pressure (mmHg) maintained at for the following brain temperatures. Tick all that apply. Tick "NA" if listed temperature is not achieved during neonatal aortic arch surgery.	Yes	No	Yes	No
27	What sites do you use routinely to measure temperature continuously during neonatal aortic arch surgery? Tick all that apply.	Yes	Yes	Yes	Yes
28	Of these sites, which is your preferred option to estimate BRAIN temperature during neonatal aortic arch surgery?	Yes	Yes	Yes	Yes
29	To what BRAIN temperature (°C) do you routinely cool prior to circulatory arrest in neonates undergoing aortic arch surgery?	Yes	Yes	Yes	Yes
30	What patient factors may change your target brain temperature prior to circulatory arrest, and if so, how?	Yes	Yes	Yes	Yes
31	Do you have a minimum cooling duration prior to circulatory arrest?	Yes	Yes	Yes	Yes
32	Yes, a minimum cooling duration before circulatory arrest- please provide total cooling duration before circulatory arrest in minutes	Yes	Yes	Yes	Yes
33	Yes, a fixed amount of time at the target brain temperature- please provide the total duration at this temperature before circulatory arrest in minutes	Yes	Yes	Yes	Yes
34	During hypothermic circulatory arrest, which of the following best reflects your routine	Yes	No	Yes	No

	perfusion practice for aortic arch surgery				
35	What is your cardiac index (L/min/m2) maintained at during the following perfusion strategies. (Please select "NA" if this perfusion strategy is not applicable to your practice).	No	No	Yes	No
36	What is your cerebral arterial blood pressure (mmHg) maintained at during the following perfusion strategies. Please tick all that apply. (Please select "NA" if this perfusion strategy is not applicable to your practice).	No	No	Yes	No
37	What is your cerebral blood temperature (°C) maintained at as part of your cerebral perfusion strategy?	No	No	Yes	No
38	With this temperature and perfusion strategy, what duration of hypothermic circulatory arrest would neonatal brain injury start to be a concern for you?	Yes	No	No	No
39	Which of the following perioperative devices are routinely used in your centre to diagnose neurological injury in neonates undergoing aortic arch surgery. Tick all that apply.	Yes	No	No	No
40	During neonatal aortic arch surgery, what haematocrit do you aim for WHILST ON CPB?	Yes	Yes	Yes	No
41	During neonatal aortic arch surgery, what haematocrit do you aim for WHEN COMING OFF CPB?	Yes	Yes	Yes	No
42	In addition to activated coagulation time (ACT), which of the following tests do you use to guide heparin anticoagulation and products given after CPB? Tick all that apply.	No	No	Yes	No
43	Do you have point of care Full Blood Count (FBC) in your centre?	No	No	Yes	No
44	Does your practice for INFANTS (31 days old – 1 year) differ from that described above for neonates?	Yes	Yes	Yes	Yes
45	How does it differ for infants? Please provide details:	Yes	Yes	Yes	Yes
46	Does your practice for OLDER CHILDREN (> 1 year – 5 years) differ from that described above for neonates?	Yes	Yes	Yes	Yes
47	How does it differ for older children? Please provide details	Yes	Yes	Yes	Yes
48	In principle, would you be interested in participating in a multi-centre clinical trial of neuromonitoring during paediatric aortic arch surgery	Yes	Yes	Yes	Yes
49	If you are happy for us to contact you regarding future research in this area, please provide your email address:	Yes	Yes	Yes	Yes

Table S2. Breakdown of survey responses from specialist groups.

Specialist group	Survey sent to, n	Responses from, n	Response rate, %
Surgeons	41	13	32%
Anaesthetists	96	17	18%
Perfusionists	13	13	100%
Neurophysiologists *	12	12	100%
**Total**	**162**	**55**	**34%**

*This group consisted of 11 Clinical Scientists in Clinical Neurophysiology and one Consultant Clinical Neurophysiologist.

Table S3. Response from each Level 1 paediatric cardiac surgical centre, by specialist group.

Centre	Surgeons	Anaesthetists	Perfusionists	Neurophysiologists	Total
Birmingham	3	3	1	1	8
Bristol	2	2	1	1	6
Dublin	1	1	2	1	5
Glasgow	1	1	1	1	4
Leeds	1	1	1	1	4
Leicester	1	1	1	1	4
Liverpool	2	2	1	1	6
London – Evelina	0	1	1	1	3
London – GOSH	1	1	1	1	4
London – RBH	0	1	1	1	3
Newcastle	1	2	1	1	5
Southampton	0	1	1	1	3
**Total**	**13**	**17**	**13**	**12**	**55**

GOSH, Great Ormond Street Hospital for Children; RBH, Royal Brompton Hospital.
